# Adherence to a diet with higher protein quality significantly reduces the risk of lung cancer: results from a population-based prospective study

**DOI:** 10.3389/fpubh.2026.1869028

**Published:** 2026-07-08

**Authors:** Ningbo Shu, Linglong Peng, Yuxiang Luo, Yaxu Wang, Dazhan Feng, Linghai Zeng, Jian Tang

**Affiliations:** 1 Gastrointestinal Ward, Department of General Surgery, Dianjiang People's Hospital of Chongqing, Chongqing, China; 2 Department of Gastrointestinal Surgery, The Second Affiliated Hospital of Chongqing Medical University, Chongqing, China; 3 Department of Cardiology, Cardiovascular Institute, Erasmus University Medical Center, Rotterdam, Netherlands; 4 Department of General Surgery, The Third Affiliated Hospital of Chongqing Medical University, Chongqing, China; 5 Department of Gastrointestinal Surgery, Chongqing Jiulongpo People's Hospital, Chongqing, China

**Keywords:** cancer prevention, cohort study, epidemiology, healthy plate protein quality index, lung cancer

## Abstract

**Background:**

The impact of protein quality on lung cancer risk in the U.S. population remains unclear. We conducted a large-scale prospective cohort study with 101,755 American adults enrolled in the Prostate, Lung, Colorectal, and Ovarian (PLCO) Cancer Screening Trial (1993–2001).

**Methods:**

The Healthy Plate Protein Quality Index (HPPQI) was used to assess dietary protein quality. Cox proportional hazards regression was applied to analyze the relationships between HPPQI and lung cancer incidence and mortality. Subgroup and sensitivity analyses were conducted.

**Results:**

Over a mean follow-up of 8.82 ± 1.95 years (897,809 person-years; median 9.40 years), 1,706 lung cancer cases (1,464 NSCLC and 242 SCLC) and 1,217 related deaths (1,005 NSCLC and 212 SCLC) were recorded. Higher HPPQI was significantly linked to lower lung cancer incidence (HR Q4 vs. Q1: 0.63; 95% CI: 0.55–0.73; *P* < 0.001 for trend) and mortality (HR Q4 vs. Q1: 0.62; 95% CI: 0.52–0.74; *P* < 0.001 for trend), consistent in both NSCLC and SCLC. Sensitivity analysis confirmed the study's robustness across various participant characteristics.

**Conclusions:**

Adherence to a dietary pattern characterized by a higher HPPQI is associated with reduced lung cancer risk.

## Introduction

Lung cancer is still the main cause of cancer-related deaths globally. It accounts for about 12.4% of all cancer diagnoses. In 2022 (1), it caused more than 1.8 million deaths worldwide (1). In the United States, projections show that there will be approximately 226,650 new cases and 124,730 deaths by 2025. This situation disproportionately affects individuals aged 50 and above (2). Although lung cancer is notably more prevalent in developing countries due to higher smoking rates and inadequate tobacco regulation (3), its causes are not limited to well-established risk factors. These include tobacco consumption, radon exposure, air pollution, occupational exposures, and genetic susceptibility (4–6). Given the intricate mechanisms underlying lung cancer development, additional potential risk factors warrant further exploration to refine preventive measures.

Modifiable lifestyle factors include diet, a well - established critical determinant of lung cancer risk (7). Protein, vital for life - sustaining processes and health promotion, is central to this risk (8). Recently, protein - lung cancer associations have become a research priority (9–11). As cells' and tissues' fundamental constituents, protein type, source, and intake affect lung cancer risk (12). However, current research on the relationship between total protein intake and the risk of CRC has yielded complex and sometimes contradictory results. Some studies have indicated an association between high - protein diets and an increased risk of CRC (13). However, we found that the proteins investigated in these studies were primarily low - quality proteins rich in harmful substances such as saturated fats, cholesterol, and heterocyclic amines. These harmful substances in the proteins can promote abnormal proliferation of intestinal cells, thereby elevating the likelihood of carcinogenesis (14). In contrast, other studies have shown that high-protein diets are mainly composed of high-quality proteins. These proteins are rich in beneficial components like fiber, antioxidants, and phytochemicals. They are closely related to a significant reduction in the risk of CRC (15, 16). Adjusting protein intake to favor high-quality proteins and reduce low-quality ones may lower lung cancer risk (17). Due to these complexities, we now assess the Healthy Plate Protein Quality Index (HPPQI) instead of solely quantifying protein intake (18).

As a comprehensive metric, the HPPQI encompasses factors like protein type, origin, amino acid profile, digestibility, and bioavailability, offering a holistic assessment of protein's nutritional and health - promoting attributes (19, 20). Calculating HPPQI enables researchers to precisely evaluate the distinct effects of various protein sources on lung cancer risk, laying a solid groundwork for developing evidence - based dietary guidelines (16). In essence, the protein - lung cancer link is intricate and multifaceted, demanding an all - encompassing evaluation approach. This approach should prioritize HPPQI - based protein quality assessment over mere protein intake quantification to fully clarify its impact on lung cancer risk. In - depth research on the relationship between HPPQI and lung cancer risk is expected to clarify their association and provide a basis for formulating more targeted and effective strategies for lung cancer prevention and management.

We conducted a large-scale, prospective study to address critical knowledge gaps and elucidate the role of protein quality, assessed via the HPPQI, in lung cancer outcomes among Americans aged 55–74. We also analyzed the two main histological subtypes, non-small cell lung cancer (NSCLC) and small cell lung cancer (SCLC), to examine subtype-specific associations. Given lung cancer's significant health and economic toll in the US, this research could inform effective prevention strategies.

## Method

### Study design and population for analysis

**Figure 1:**
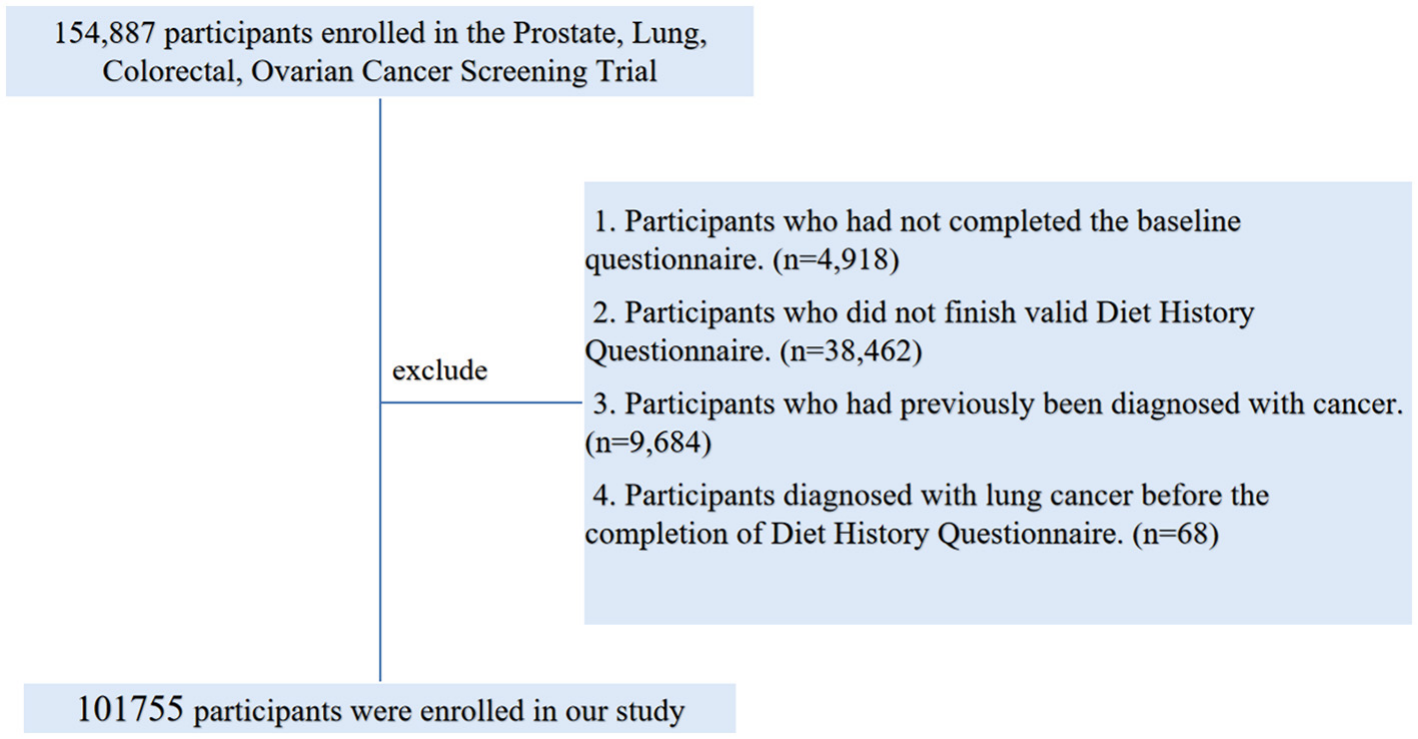
The flow chart of identifying eligible subjects. PLCO, Prostate, Lung, Colorectal, and Ovarian; BQ, Baseline Questionnaire; DHQ, Diet History Questionnaire.

The PLCO cancer screening trial aimed to determine if certain cancer screening modalities could lower the incidence of prostate, lung, colorectal, and ovarian cancers (1). Between November 1993 and September 2001, 154,887 adults aged 55–74 were enrolled across 10 strategically located US screening centers (21). Participants were randomized to either a screening group (receiving routine screenings for the four cancers) or a control group (continuing standard medical care). Demographic data were mainly collected via the Baseline Questionnaire (BQ), and dietary habits were assessed using the Dietary History Questionnaire (DHQ). The PLCO trial protocol was approved by the NCI's and participating centers' institutional review boards, and all participants gave informed written consent. At baseline, we applied several exclusion criteria to determine the final analytic sample: (1) Participants who had not completed the Baseline Questionnaire (*n* = 4,918); (2) Participants who did not finished valid Diet History Questionnaire (those failure to return DHQ responses, those lacking a completion date, those completed after the death date, those with a high frequency of missing responses (≥8), or those with extremely high energy intake values (the first or last percentile)) (*n* = 38,462); (3) Participants who had previously been diagnosed with cancer (*n* = 9,684); (4) Participants diagnosed with lung cancer before the completion of Diet History Questionnaire (*n* = 68). Ultimately, our analytic sample consisted of 101,755 individuals (49,496 males and 52,259 females) (Figure 1).

### Data collection and covariates assessment

Demographic data for the PLCO trial were gathered via self - administered BQ. Comprehensive baseline characteristics were recorded, encompassing age, gender, race, marital status, education, trial arm allocation, family cancer history, and histories of hypertension, chronic bronchitis, emphysema, diabetes, etc. Anthropometric measures included body mass index (BMI, calculated as weight [kg] / height^2^ [m^2^]) and weight change (difference between baseline and self - reported weight at age 20). Smoking status was assessed using two parameters: smoking history (never, or current/former) and intensity (0, 1–20, or >20 cigarettes/day).

### Dietary assessment

Dietary information was collected only once at baseline using the Diet History Questionnaire (DHQ), a self-administered Food Frequency Questionnaire (FFQ) administered post-enrollment. The 137-item DHQ systematically evaluated dietary intake over the past year, including frequencies of various food groups (meats, vegetables, fruits), supplement use, and portion sizes. Validation studies have demonstrated the DHQ's excellent performance in nutrient intake assessment, confirming its reliability and validity for capturing long-term dietary patterns in large-scale epidemiological studies (22). The HPPQI assesses protein source quality using a ratio that favors seafood, poultry, pulses, and nuts—sources abundant in high-quality protein, essential amino acids, and bioactive compounds—over red/processed meats and cheese (including beef, pork, lamb, and their cuts; processed meats such as bacon, sausage, hot dogs, ham, cold cuts, and other cured, fermented, smoked, or otherwise processed meats; and various types of cheese (23, 24). This aligns with global dietary guidelines promoting plant - based protein choices. The HPPQI is computed based on the following ratio (25):

*HPPQI* = *(seafood*+*poultry*+*pulses*+*nuts) / (red and processed meats*+*cheese)*

Therefore, the HPPQI serves as an indicator of a broader dietary pattern, reflecting the relative consumption of preferred protein sources vs. red/processed meats and cheese.

### Ascertainment of outcome events

In the PLCO trial, lung cancer case detection mainly relied on the annual update system. Participants provided comprehensive cancer data (diagnosis type, date, facility, and physician contacts). All reported cases were verified via medical records, and vital status was tracked through annual forms. For non - responders, the team followed up by phone/email. For confirmation, the diagnostic coding system for cancer cases was ICD-O-2, while cause-of-death data were derived from death certificates coded with ICD-9, in which the specific diagnostic code for lung cancer is 162XX (encompassing all histological subtypes per ICD-9 conventions).

### Statistical analysis

The study contains several variables with missing data. For covariates with missing values < 5%, categorical variables including race, marital status, educational level, history of aspirin use, history of diabetes, emphysema, chronic bronchitis and hypertension, history of X-ray exposure, family history of lung cancer, smoking status and so on were imputed using modal values. Continuous covariates with missing values < 5%, specifically BMI and pack-years of smoking, were imputed using median values (26). The detailed imputation information for each missing data item and its corresponding proportion is presented in Supplementary Table 1.

**Figure 2:**
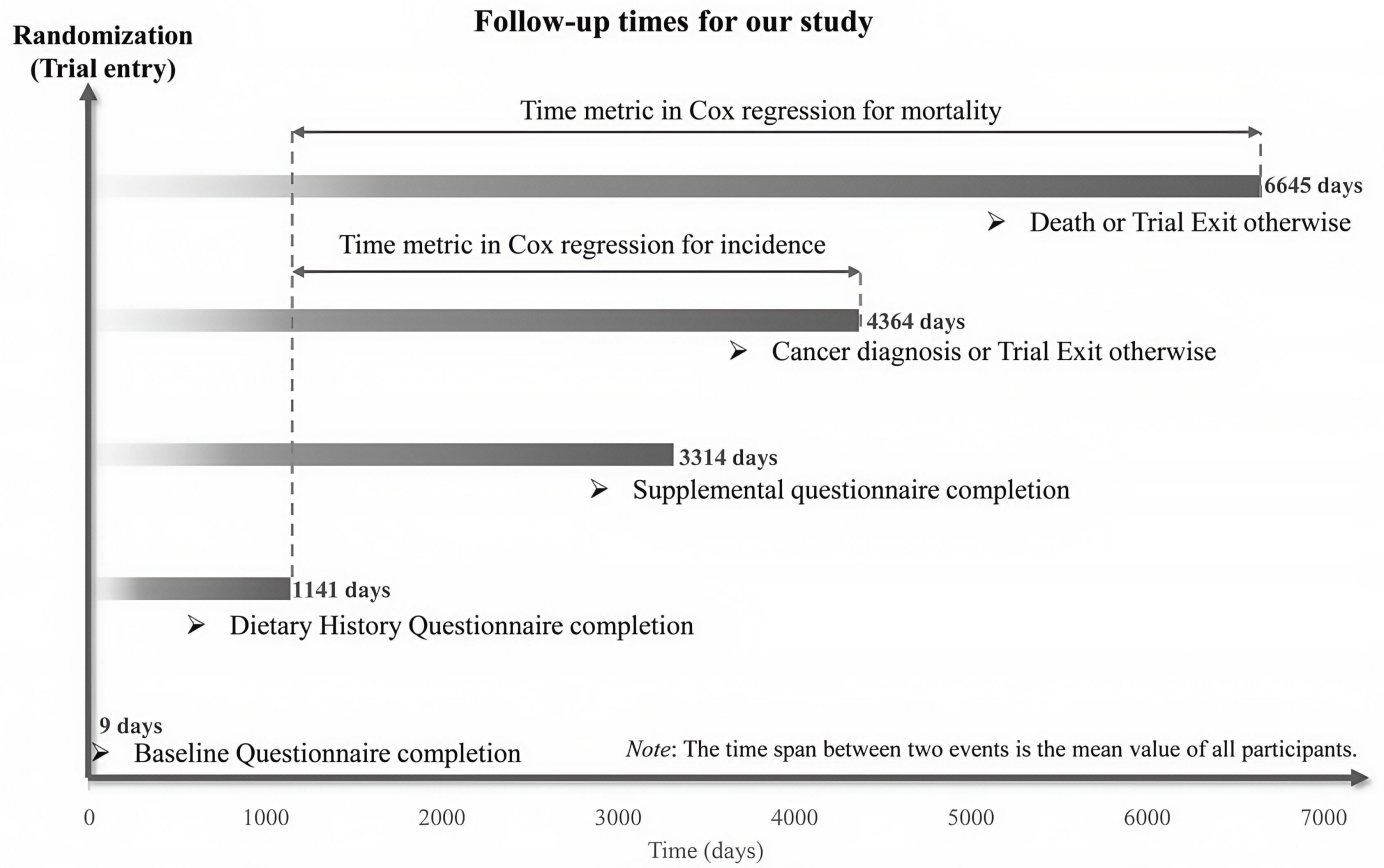
The timeline and follow-up scheme of our study.

In this study, time - to - lung cancer event (diagnosis or related death) was calculated as days from DHQ completion until lung cancer diagnosis or confirmation of lung - cancer - related death. For primary outcomes, follow - up duration was measured from DHQ completion to the first occurrence of lung cancer diagnosis, death, loss to follow - up, or December 31, 2009 (end of cancer incidence follow - up). For secondary outcomes, mortality follow - up ended in 2018, as detailed on the PLCO website (https://cdas.cancer.gov/learn/plco/early-qx/) (Figure 2). Cox proportional hazards regression models were built to estimate hazard ratios (HRs) and 95% confidence intervals (CIs) for HPPQI - outcome associations, using follow - up time as the metric. HPPQI was analyzed as a categorical variable, stratified into quartiles with the first quartile as the reference. To assess linear trends, variables were constructed using the median HPPQI values within each quartile (i.e., quantile-based median values), and the *P*-value indicated the significance of linear trends. Potential confounders were selected based on established lung cancer risk factors or investigators' clinical expertise (27) and included in the Cox models. Model 1 adjusted for demographic factors (sex, age, race, education, marital status). Model 2 additionally adjusted for lifestyle and clinical factors (BMI, smoking status, daily cigarettes consumption, hypertension, emphysema/chronic bronchitis history, aspirin use, diabetes, family lung cancer history) and trial group. We employed restricted cubic spline (RCS) models to depict HPPQI - lung cancer incidence/mortality links, with median HPPQI as reference. Nonlinearity was tested by checking if the second - spline - term's regression coefficient was zero (28). Similar analyses were conducted for NSCLC and SCLC.

Prespecified subgroup analyses assessed potential effect modification of the HPPQI - lung cancer incidence link by key factors. Subgroups were defined by demographic (age >65 vs. ≤ 65, sex male vs. female, race White vs. non - White, marital status married vs. unmarried), health (diabetes yes/no, hypertension yes/no, baseline BMI ≤ 30 vs. >30 kg/m^2^), family/medical history (lung cancer family history absent/present, emphysema history yes/no, chronic bronchitis history yes/no), and lifestyle (smoking status never vs. current/former, aspirin use no vs. yes, daily cigarettes 0 vs. 1–20 vs. >20) characteristics. Interaction *P*-values were calculated by comparing models with and without interaction terms to detect potential spurious subgroup effects.

To bolster the robustness of results, we performed sensitivity analyses (27, 29, 30):

(1) To address potential reverse causality, we conducted sensitivity analyses by sequentially excluding cases occurring within the first two and 4 years of follow-up.

(2) We excluded participants with extreme energy intake (energy intake >4,000 kcal/day or < 500 kcal/day).

(3) We excluded participants with extreme BMI values (below the 1st percentile and above the 99th percentile) to minimize the influence of outliers.

(4) To improve the statistical power of the study, pack-years of smoking were adjusted instead of daily cigarette consumption (ranging from 0 to 20).

(5) Given the elevated risk of lung cancer associated with certain medical conditions, we excluded individuals with diabetes mellitus and respiratory comorbidities, including emphysema and chronic bronchitis.

(6) We further adjusted for alcohol consumption history, dietary fiber intake, and fruit and vegetable intake.

(7) As a ratio variable, HPPQI inherently possesses continuous characteristics, justifying its analysis as a continuous variable.

All statistical analyses were conducted in R (version 4.3.1), with a two - tailed *P* < 0.05 indicating statistical significance.

## Result

### Participant baseline features

In this study, the median HPPQI among participants was 0.91, with an interquartile range (IQR) of 1.13. Participants were categorized into quartiles based on their HPPQI: Q1 (0–0.54; median = 0.37), Q2 (>0.54–0.91; median = 0.71), Q3 (>0.91–1.67; median = 1.20), and Q4 (>1.67; median = 2.76). As shown in Table 1, those in Q4 (the highest quartile) were more prone to being female, non - white, and highly educated, but less likely to be married, aspirin users, or diagnosed with diabetes, hypertension, or emphysema. Additionally, participants in Q4 were less likely to have a family history of lung cancer, lower smoking intensity, and a lower, less variable BMI.

**Table 1:** Baseline characteristics of study population according to HPPQI.

Characteristics	Overall	Quartiles of overall HPPQI				*P* overall
		Quartile 1	Quartile 2	Quartile 3	Quartile 4	
Number of participants	10,1755	25,439	25,439	25,438	25,439	–
Age	62.40 ± 5.28	62.10 ± 5.18	62.36 ± 5.24	62.51 ± 5.29	62.65 ± 5.41	< 0.001
Sex						< 0.001
Male	49,496 (48.64%)	17,482 (68.72%)	13,393 (52.65%)	10,520 (41.36%)	8,101 (31.84%)	
Female	52,259 (51.36%)	7,957 (31.28%)	12,046 (47.35%)	14,918 (58.64%)	17,338 (68.16%)	
Race						< 0.001
White	94,066 (92.44%)	24,369 (95.79%)	23,968 (94.22%)	23,573 (92.67%)	22,156 (87.09%)	< 0.001
Non-white	7,689 (7.56%)	1,070 (4.21%)	1,471 (5.78%)	1,865 (7.33%)	3,283 (12.91%)	
Education level						< 0.001
College below	64,953 (63.83%)	18,096 (71.13%)	16,558 (65.09%)	15,759 (61.95%)	14,540 (57.16%)	
College graduate	17,848 (17.54%)	3,918 (15.40%)	4,415 (17.36%)	4,694 (18.45%)	4,821 (18.95%)	
Postgraduate	18,954 (18.63%)	3,425 (13.46%)	4,466 (17.56%)	4,985 (19.60%)	6,078 (23.89%)	
Marriage						< 0.001
Married	79,826 (78.45%)	20,451 (80.39%)	20,679 (81.29%)	20,007 (78.65%)	18,689 (73.47%)	
Unmarried	21,929 (21.55%)	4,988 (19.61%)	4,760 (18.71%)	5,431 (21.35%)	6,750 (26.53%)	
Diabetes history						< 0.001
No	94,949 (93.31%)	23,497 (92.37%)	23,694 (93.14%)	23,811 (93.60%)	23,947 (94.13%)	
Yes	6,806 (6.69%)	1,942 (7.63%)	1,745 (6.86%)	1,627 (6.40%)	1,492 (5.87%)	
Aspirin use history						< 0.001
No	53,953 (53.02%)	13,179 (51.81%)	13,339 (52.44%)	13,418 (52.75%)	14,017 (55.10%)	< 0.001
Yes	47,802 (46.98%)	12,260 (48.19%)	12,100 (47.56%)	12,020 (47.25%)	11,422 (44.90%)	
X-ray history						0.070
No	46,303 (45.51%)	11,396 (44.81%)	11,611 (45.62%)	11,716 (46.05%)	11,580 (45.51%)	
Once	32,918 (32.35%)	8,339 (32.77%)	8,241 (32.39%)	8,123 (31.93%)	8,215 (32.30%)	
More than once	18,377(18.06%)	4,709 (18.51%)	4,566 (17.99%)	4,499 (17.70%)	4,603 (18.10%)	
Possibly	4,157(4.08%)	995 (3.91%)	1,021 (4.00%)	1,100 (4.32%)	1,041 (4.09%)	
Family history of lung cancer						< 0.001
No	88,738 (87.21%)	22,023 (86.57%)	22,190 (87.23%)	22,251 (87.47%)	22,274 (87.56%)	
Yes	10,569 (10.39%)	2,679 (10.53%)	2,643 (10.39%)	2,598 (10.21%)	2,649 (10.41%)	
Possibly	2,448 (2.41%)	737 (2.90%)	606 (2.38%)	589 (2.32%)	516 (2.03%)	
Chronic bronchitis history						0.057
No	97,423 (95.74%)	24,299 (95.52%)	24,386 (95.86%)	24,409 (95.95%)	24,329 (95.64%)	0.057
Yes	4,332 (4.26%)	1,140 (4.48%)	1,053 (4.14%)	1,029 (4.05%)	1,110 (4.36%)	
Emphysema history						< 0.001
No	99,611 (97.89%)	24,670 (96.98%)	24,877 (97.79%)	24,989 (98.23%)	25,075 (98.57%)	
Yes	2,144 (2.11%)	769 (3.02%)	562 (2.21%)	449 (1.77%)	364 (1.43%)	
Hypertension history						< 0.001
No	68,707 (67.52%)	16,894 (66.41%)	17,012 (66.87%)	17,199 (67.61%)	17,602 (69.19%)	
Yes	33,048 (32.48%)	8,545 (33.59%)	8,427 (33.13%)	8,239 (32.39%)	7,837 (30.81%)	
Family history of cancer						< 0.001
No	44,899 (44.12%)	11,530 (45.32%)	11,142 (43.80%)	11,235 (44.17%)	10,992 (43.21%)	
Yes	56,856 (55.88%)	13,909 (54.68%)	14,297 (56.20%)	14,203 (55.83%)	14,447 (56.79%)	
Arm						0.208
Intervention	51,817 (50.92%)	12,849 (50.51%)	12,976 (51.01%)	13,079 (51.42%)	12,913 (50.76%)	
Control	49,938 (49.08%)	12,590 (49.49%)	12,463 (48.99%)	12,359 (48.58%)	12,526 (49.24%)	
Smoking status						< 0.001
No	48,580 (47.74%)	10,013 (39.36%)	11,683 (45.93%)	13,069 (51.38%)	13,815 (54.31%)	
Current/former	53,175 (52.26%)	15,426 (60.64%)	13,756 (54.07%)	12,369 (48.62%)	11,624 (45.69%)	
Body mass index at baseline (kg/m^2^)	27.22 ± 4.79	27.98 ± 4.72	27.56 ± 4.74	27.10 ± 4.72	26.26 ± 4.78	< 0.001
Weight fluctuation^a^	2.84 ± 0.82	2.98 ± 0.82	2.90 ± 0.82	2.82 ± 0.82	2.68 ± 0.82	< 0.001
Smoking pack-years	17.65 ± 26.59	24.26 ± 31.22	18.50 ± 26.69	15.33 ± 24.43	12.52 ± 21.62	< 0.001
Daily cigarette consumption						< 0.001
0	48,685 (47.85%)	10,053 (39.52%)	11,701 (46.00%)	13,091 (51.46%)	13,840 (54.40%)	
1–20	33,218 (32.65%)	8,395 (33.00%)	8,519 (33.49%)	8,092 (31.81%)	8,212 (32.28%)	
>20	19,852 (19.51%)	6,991 (27.48%)	5,219 (20.52%)	4,255 (16.73%)	3,387 (13.31%)	

Descriptive statistics are presented as (mean ± standard deviation) and number (percentage) for continuous and categorical.

^a^Weight fluctuation was defined as the participant's baseline weight minus weight at age 20.

### Association between lung cancer incidence and HPPQI

**Figure 3:**
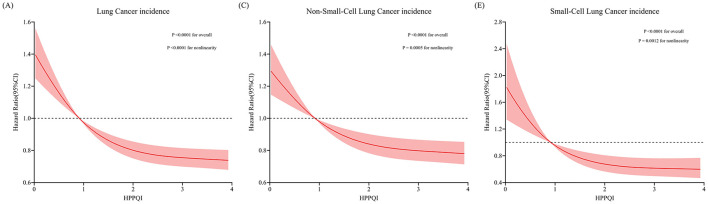
RCS model on the association of HPPQI with the lung cancer incidence. Hazard ratio was adjusted for age (years), sex (male, female), race (white and non-white), education levels (college below, college graduate, postgraduate), marital status (married, unmarried), smoking status (never, currently/ever), number of cigarettes smoked (0, 1–20, > 20 cigarettes/day), history of emphysema (yes, no), chronic bronchitis history (yes, no), body mass index (kg/m^2^), trail arm (intervention, control), aspirin use (yes, no), history of diabetes (yes, no), history of hypertension (yes, no) and family history of lung cancer (yes, no).

During an average follow - up of 8.82 ± 1.95 years (897,809 person - years), 1,706 cases were documented, comprising 1,464 NSCLCs and 242 SCLCs. This yielded an overall incidence rate of roughly 19.00 cases per 10,000 person - years. As illustrated in Table 2, cox regression analysis showed a significant inverse association between higher HPPQI and lung cancer incidence after adjusting for potential confounders. (HR Q4 vs. Q1: 0.63; 95% CI: 0.55, 0.73. *P* < 0.001 for trend). Similar negative correlations were observed in the association between HPPQI and the incidence of NSCLC (HR Q4 vs. Q1: 0.68; 95% CI: 0.58, 0.79; *P* < 0.001 for trend) and SCLC incidence (HR Q4 vs. Q1: 0.39; 95% CI: 0.25, 0.61; *P* < 0.001 for trend). The RCS model revealed nonlinear relationships between HPPQI and the incidence of overall lung cancer and both NSCLC and SCLC (Figure 3).

**Table 2:** Hazard ratios of the association between HPPQI and lung cancer incidence.

Quartiles of HPPQI	Cases	Person-years	Incidence rate per 10,000 person-years (95% confidence interval)	Hazard ratio (95% confidence interval) by HPPQI		
				Unadjusted	Model 1^a^	Model 2^b^
Lung cancer						
Quartile 1	616	220,890.5	27.89 (25.77, 30.17)	1.000 (reference)	1.000 (reference)	1.000 (reference)
Quartile 2	415	223,968.3	18.53 (16.83, 20.40)	0.66 (0.59, 0.75)	0.73 (0.64, 0.82)	0.77 (0.68, 0.88)
Quartile 3	378	225,520.7	16.76 (15.16, 18.54)	0.60 (0.53, 0.68)	0.68 (0.60, 0.78)	0.77 (0.68, 0.88)
Quartile 4	297	227,429.9	13.06 (11.66, 14.63)	0.47 (0.41, 0.54)	0.56 (0.48, 0.64)	0.63 (0.55, 0.73)
*p* for trend	–	–	–	< 0.001	< 0.001	< 0.001
Non-small-cell lung cancer						
Quartile 1	517	220, 890.5	23.41 (21.47, 25.51)	1.000 (reference)	1.000 (reference)	1.000 (reference)
Quartile 2	347	223, 968.3	15.49 (13.95, 17.21)	0.66 (0.58, 0.76)	0.72 (0.63, 0.83)	0.77 (0.67, 0.88)
Quartile 3	330	225, 520.7	14.63 (13.14, 16.30)	0.62 (0.54, 0.72)	0.71 (0.62, 0.82)	0.80 (0.69, 0.92)
Quartile 4	270	227, 429.9	11.87 (10.54, 13.37)	0.51 (0.44, 0.59)	0.60 (0.51, 0.70)	0.68 (0.58, 0.79)
*p* for trend	–	–	–	< 0.001	< 0.001	< 0.001
Small-cell lung cancer						
Quartile 1	99	220, 890.5	4.48 (3.68, 5.46)	1.000 (reference)	1.000 (reference)	1.000 (reference)
Quartile 2	68	223, 968.3	3.04 (2.40, 3.85)	0.68 (0.50, 0.92)	0.75 (0.55, 1.02)	0.81 (0.59, 1.10)
Quartile 3	48	225, 520.7	2.13 (1.61, 2.82)	0.47 (0.34, 0.67)	0.55 (0.39, 0.78)	0.64 (0.45, 0.91)
Quartile 4	27	227, 429.9	1.19 (0.82, 1.73)	0.26 (0.17, 0.40)	0.33 (0.21, 0.51)	0.39 (0.25, 0.61)
*p* for trend	–	–	–	< 0.001	< 0.001	< 0.001

^a^Model 1 was adjusted with age (continuous), sex (male, female), race (white, non-white), education levels (college below, college graduate, postgraduate) and marriage (married, unmarried).

^b^Model 2 was adjusted for model 1 plus BMI at baseline (continuous), trail arm (intervention, control), smoking status (never, current or former), daily cigarette consumption (0, 1–20, >20), aspirin use (no, yes), family history of lung cancer (no, yes, possibly), history of hypertension (no, yes), history of diabetes (no, yes), history of chronic bronchitis (no, yes), history of emphysema (no, yes).

Subgroup analysis revealed a significant interaction (*P* < 0.05) between age and emphysema history in modulating the negative association between HPPQI and lung cancer incidence. This finding suggests that a higher HPPQI exerts a stronger protective effect in reducing the incidence of lung cancer among individuals aged 55–65 years (HR Q4 vs. Q1: 0.52; 95% CI: 0.43, 0.63; *P* < 0.001 for trend) compared to those aged 65–74 years (HR Q4 vs. Q1: 0.73; 95% CI: 0.58, 0.91; *P* = 0.009 for trend). Similarly, the protective effect of a higher HPPQI is more pronounced in individuals without a history of emphysema (HR Q4 vs. Q1: 0.58; 95% CI: 0.50, 0.68; *P* < 0.001 for trend) compared to those with a history of emphysema (HR Q4 vs. Q1: 0.56; 95% CI: 0.31, 0.99; *P* = 0.034 for trend). In contrast, no significant differences in the negative association between HPPQI and lung cancer incidence were observed across other subgroups (all interaction *P*-values > 0.05), as detailed in Supplementary Table 2. Furthermore, sensitivity analyses robustly supported the relationship between HPPQI and lung cancer incidence (Tables 3-1, 3-2).

**Table 3-1:** The sensitivity analyses between HPPQI and lung cancer incidence.

Categories	HR^e^ (Quartile 4 vs. Quartile 1, 95% CI)	*p* for trend
Exclude extreme energy intake^a^	0.64 (0.54, 0.75)	< 0.001
Exclude extreme BMI^b^	0.63 (0.54, 0.73)	< 0.001
Excluding Patients diagnosed within 2 years	0.57 (0.49, 0.68)	< 0.001
Excluding Patients diagnosed within 4 years	0.53 (0.44, 0.64)	< 0.001
Excluding patients with diabetes or respiratory comorbidities^c^	0.64 (0.55, 0.75)	< 0.001
Replace the Indicator of cigarettes smoked^d^	0.70 (0.61, 0.81)	< 0.001
Further adjusted for alcohol history, dietary fiber, and fruit/vegetable intake	0.71 (0.61, 0.83)	< 0.001

^a^Extreme energy intake was defined as energy intake >4,000 kcal/day or < 500 kcal/day.

^b^BMI defined as body mass index at baseline (kg/m2).

^c^Respiratory comorbidities included emphysema and chronic bronchitis.

^d^Adjusting for pack-years of smoking (continuous) instead of daily cigarette consumption (0, 1–20, or >20).

^e^HR was adjusted for age (years), sex (male, female), race (white and non-white), education levels (college below, college graduate, postgraduate), marital status (married, unmarried), smoking status (never, currently/ever), number of cigarettes smoked (0, 1–20, > 20 cigarettes/day), history of emphysema (yes, no), chronic bronchitis history (yes, no), body mass index (kg/m2), trail arm (intervention, control), aspirin use (yes, no), history of diabetes (yes, no), history of hypertension (yes, no) and family history of lung cancer (yes, no).

**Table 3-2:** The sensitivity analyses between HPPQI and lung cancer incidence.

Analyzing HPPQI as a continuous variable	HR (95% CI)		
	Unadjusted	Model 1	Model 2
Lung cancer	0.92 (0.90, 0.95)	0.95 (0.93, 0.97)	0.96 (0.94, 0.98)
*P*-value	< 0.001	< 0.001	0.001
Non-small-cell lung cancer	0.94 (0.91, 0.96)	0.96 (0.93, 0.98)	0.97 (0.94, 0.99)
*P*-value	< 0.001	0.001	0.003
Small-cell lung cancer	0.80 (0.71, 0.91)	0.86 (0.77, 0.96)	0.89 (0.80, 0.99)
*P*-value	< 0.001	0.008	0.028

### Association between lung cancer mortality and HPPQI

**Figure 4:**
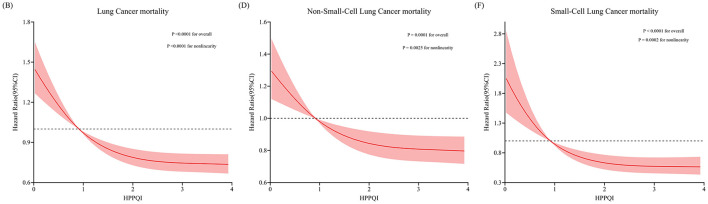
RCS model on the association of HPPQI with the lung cancer mortality. Hazard ratio was adjusted for age (years), sex (male, female), race (white and non-white), education levels (college below, college graduate, postgraduate), marital status (married, unmarried), smoking status (never, currently/ever), number of cigarettes smoked (0, 1–20, > 20 cigarettes/day), history of emphysema (yes, no), chronic bronchitis history (yes, no), body mass index (kg/m^2^), trail arm (intervention, control), aspirin use (yes, no), history of diabetes (yes, no), history of hypertension (yes, no) and family history of lung cancer (yes, no).

During an average follow - up of 15.07 ± 4.54 years (1,533,359 person - years), 1,217 deaths attributed to lung cancer were documented, comprising 1,005 cases of NSCLCs and 212 cases of SCLCs. This yielded an overall mortality rate of roughly 7.94 deaths per 10,000 person - years.Table 4 presented the results of multivariable Cox regression analysis examining the association between HPPQI and lung cancer mortality. Similar to the association between HPPQI and lung cancer incidence, the multivariable adjusted model showed a lower risk of lung cancer mortality among individuals in the highest HPPQI quartile, compared to the lowest HPPQI quartile (HR Q4 vs. Q1: 0.62; 95% CI: 0.52, 0.74; *p* < 0.001 for trend). Similar negative correlations were observed in the relationships between HPPQI and NSCLC mortality (HR Q4 vs. Q1: 0.69; 95% CI: 0.57, 0.84; *p* = 0.001 for trend) as well as between HPPQI and SCLC mortality (HR Q4 vs. Q1: 0.33; 95% CI: 0.21, 0.54; *p* < 0.001 for trend). The RCS model revealed nonlinear relationships between HPPQI and the mortality of overall lung cancer and both NSCLC and SCLC (Figure 4).

**Table 4:** Hazard ratios of the association between HPPQI and lung cancer mortality.

Quartiles of HPPQI	Cases	Person-years	Incidence rate per 10,000 person-years (95% confidence interval)	Hazard ratio (95% confidence interval) by HPPQI		
				Unadjusted	Model 1^a^	Model 2^b^
Lung cancer						
Quartile 1	440	369, 643.9	11.90 (10.84, 13.07)	1.000 (reference)	1.000 (reference)	1.000 (reference)
Quartile 2	308	380, 296.5	8.10 (7.24, 9.05)	0.69 (0.59, 0.80)	0.77 (0.66, 0.89)	0.82 (0.70, 0.95)
Quartile 3	268	387, 856.2	6.91 (6.13, 7.79)	0.59 (0.51, 0.69)	0.69 (0.59, 0.81)	0.78 (0.67, 0.92)
Quartile 4	201	395, 562.4	5.08 (4.43, 5.83)	0.44 (0.37, 0.52)	0.55 (0.46, 0.65)	0.62 (0.52, 0.74)
*p* for trend				< 0.001	< 0.001	< 0.001
Non-small-cell lung cancer						
Quartile 1	351	369, 643.9	9.50 (8.55, 10.54)	1.000 (reference)	1.000 (reference)	1.000 (reference)
Quartile 2	246	380, 296.5	6.47 (5.71, 7.33)	0.69 (0.58, 0.81)	0.77 (0.65, 0.91)	0.82 (0.70, 0.97)
Quartile 3	229	387, 856.2	5.90 (5.19, 6.72)	0.63 (0.53, 0.75)	0.75 (0.63, 0.89)	0.84 (0.71, 1.00)
Quartile 4	179	395, 562.4	4.53 (3.91, 5.24)	0.49 (0.41, 0.58)	0.61 (0.51, 0.74)	0.69 (0.57, 0.84)
*p* for trend	–	–	–	< 0.001	< 0.001	0.001
Small-cell lung cancer						
Quartile 1	89	369, 643.9	2.41 (1.96, 2.96)	1.000 (reference)	1.000 (reference)	1.000 (reference)
Quartile 2	62	380, 296.5	1.63 (1.27, 2.09)	0.69 (0.50, 0.95)	0.75 (0.54, 1.04)	0.80 (0.58, 1.11)
Quartile 3	39	387, 856.2	1.01 (0.74, 1.37)	0.43 (0.29, 0.62)	0.48 (0.33, 0.71)	0.55 (0.38, 0.81)
Quartile 4	22	395, 562.4	0.56 (0.37, 0.84)	0.24 (0.15, 0.38)	0.28 (0.17, 0.46)	0.33 (0.21, 0.54)
*p* for trend	–	–	–	< 0.001	< 0.001	< 0.001

^a^Model 1 was adjusted with age (continuous), sex (male, female), race (white, non-white), education levels (college below, college graduate, postgraduate) and marriage (married, unmarried).

^b^Model 2 was adjusted for model 1 plus BMI at baseline (continuous), trail arm (intervention, control), smoking status (never, current or former), daily cigarette consumption (0, 1–20, >20), aspirin use (no, yes), family history of lung cancer (no, yes, possibly), history of hypertension (no, yes), history of diabetes (no, yes), history of chronic bronchitis (no, yes), history of emphysema (no, yes).

Subgroup analysis revealed a significant interaction (*P* < 0.05) between age and emphysema history in modulating the negative association between HPPQI and lung cancer mortality. This finding suggests that a higher HPPQI exerts a stronger protective effect in reducing the mortality of lung cancer among individuals aged 55–65 years (HR Q4 vs. Q1: 0.50; 95% CI: 0.40, 0.64; *P* < 0.001 for trend) compared to those aged 65–74 years (HR Q4 vs. Q1: 0.73; 95% CI: 0.56, 0.95; *P* = 0.009 for trend). Similarly, the protective effect of a higher HPPQI is more pronounced in individuals without a history of emphysema (HR Q4 vs. Q1: 0.58; 95% CI: 0.48, 0.69; *P* < 0.001 for trend) compared to those with a history of emphysema (HR Q4 vs. Q1: 0.39; 95% CI: 0.18, 0.85; *P* = 0.013 for trend). Contrastingly, across other subgroups, the negative relationship between HPPQI and lung cancer mortality failed to demonstrate significant inter - subgroup variations (all interaction *P*-values > 0.05), as detailed in Supplementary Table 3. Sensitivity analyses demonstrated a significant robust negative association between HPPQI value and lung cancer mortality rates (Tables 5-1, 5-2).

**Table 5-1:** The sensitivity analyses between HPPQI and lung cancer mortality.

Categories	HR^e^ (Quartile 4 vs. Quartile 1, 95% CI)	*p* for trend
Exclude extreme energy intake^a^	0.65 (0.53, 0.79)	< 0.001
Exclude extreme BMI^b^	0.61 (0.51, 0.73)	< 0.001
Excluding Patients diagnosed within 2 years	0.59 (0.49, 0.70)	< 0.001
Excluding Patients diagnosed within 4 years	0.58 (0.47, 0.71)	< 0.001
Excluding patients with diabetes or respiratory comorbidities^c^	0.65 (0.53, 0.79)	< 0.001
Replace the Indicator of cigarettes smoked^d^	0.69 (0.57, 0.82)	< 0.001
Further adjusted for alcohol history, dietary fiber, and fruit/vegetable intake	0.67 (0.56, 0.81)	< 0.001

^a^Extreme energy intake was defined as energy intake >4,000 kcal/day or < 500 kcal/day.

^b^BMI defined as body mass index at baseline (kg/m2).

^c^Respiratory comorbidities included emphysema and chronic bronchitis.

^d^Adjusting for pack-years of smoking (continuous) instead of daily cigarette consumption (0, 1–20, or >20).

^e^HR was adjusted for age (years), sex (male, female), race (white and non-white), education levels (college below, college graduate, postgraduate), marital status (married, unmarried), smoking status (never, currently/ever), number of cigarettes smoked (0, 1–20, > 20 cigarettes/day), history of emphysema (yes, no), chronic bronchitis history (yes, no), body mass index (kg/m2), trail arm (intervention, control), aspirin use (yes, no), history of diabetes (yes, no), history of hypertension (yes, no) and family history of lung cancer (yes, no).

**Table 5-2:** The sensitivity analyses between HPPQI and lung cancer mortality.

Analyzing HPPQI as a continuous variable	HR (95% CI)		
	Unadjusted	Model 1	Model 2
Lung cancer	0.92 (0.89, 0.96)	0.95 (0.93, 0.98)	0.96 (0.94, 0.99)
*P*-value	< 0.001	0.001	0.007
Non-small-cell lung cancer	0.94 (0.91, 0.97)	0.96 (0.94, 0.99)	0.97 (0.95, 0.997)
*P*-value	< 0.001	0.011	0.029
Small-cell lung cancer	0.80 (0.70, 0.91)	0.85 (0.75, 0.96)	0.88 (0.78, 0.99)
*P*-value	0.001	0.009	0.028

## Discussion

Leveraging data from the PLCO trial, this investigation assessed the linkage between HPPQI and lung cancer (both incidence and mortality). A cohort of 101,755 US adults was recruited from the PLCO trial. Elevated HPPQI were tied to a lower risk of lung cancer incidence, covering NSCLC and SCLC. Likewise, a comparable inverse pattern emerged for HPPQI and lung cancer - related mortality. It is important to recognize that the HPPQI, as a ratio, inherently reflects a broader dietary pattern rather than the isolated effect of protein quality. These findings retained statistical significance following thorough adjustment for potential confounding factors. The RCS analysis unveiled non - linear inverse trends in the incidence - mortality nexus for overall lung cancer and its NSCLC/SCLC subtypes. Sensitivity tests validated the reliability of our results.

Subgroup analysis in this study showed a significant interaction (*P* < 0.05) between age and emphysema history in affecting the link between HPPQI and lung cancer risk. This means age and emphysema history are effect modifiers, changing the strength of the HPPQI - lung cancer link, not just confounding factors (31). The interaction reveals a complex biological mechanism. HPPQI has different effects on reducing lung cancer incidence and mortality in different subgroups. For example, in some age groups (like the older adult(s)) or people with emphysema, the HPPQI - lung cancer relationship may vary due to multiple factors. Cumulative exposure effects may make different age groups respond differently to protein quality (32). Pathophysiological differences, such as physical function decline in the older adult(s) and lung tissue changes in emphysema patients, can affect protein metabolism and actions in the body (33, 34). Also, disease progression differences may lead to varying inhibitory effects of high - quality proteins on lung cancer development (35). Future investigations should employ molecular epidemiological methods (e.g., multi-omics profiling) to unravel the biological mechanisms underlying this interaction and construct dynamic risk prediction models that incorporate interaction terms. Such models would enhance our understanding of the associations relevant to lung cancer prevention and serve as a foundation for formulating more targeted public health intervention strategies.

The following mechanisms are biologically plausible based on existing literature, but none were directly tested in the present study. They should be interpreted as hypothetical explanations for the observed associations, not as demonstrated effects. Emerging evidence shows a complex, nonlinear link between protein intake and lung cancer risk, driven by biological mechanisms and overall dietary patterns (16, 36). Adequate intake of protein sources that contribute to a higher HPPQI may potentially reduce lung cancer risk through several hypothesized pathways. It provides essential amino acids (like branched - chain amino acids) for alveolar repair, enhances pulmonary antioxidant defenses with vitamins and minerals (such as vitamin D and selenium), and reduces pulmonary inflammation from tobacco smoke or air pollution with anti - inflammatory components (like ω - 3 polyunsaturated fatty acids) (37, 38). On the contrary, long - term excessive intake of animal - derived proteins, especially from processed and red meats, can raise lung cancer risk through two mechanisms. First, carcinogens (like nitrites and polycyclic aromatic hydrocarbons) from food processing directly damage DNA in respiratory epithelial cells. Second, altered bile acid metabolism and gut microbiota dysbiosis indirectly worsen chronic pulmonary inflammation (13, 14). Plant-based protein sources (such as legumes and whole grains) generally have a lower carcinogen load and are rich in dietary fiber, which helps accelerate the excretion of carcinogens and reduce systemic inflammation levels, thereby potentially reducing the incidence of lung cancer (15, 16).

Notably, recent studies suggest differences in the effects of animal- and plant-based proteins on lung cancer risk and mortality, with plant protein intake often associated with a lower risk, while certain animal proteins (such as those from red and processed meats) linked to an increased risk. This may be related to their accompanying components (such as saturated fat, heme iron, dietary fiber, and antioxidants) as well as their regulatory effects on pro-inflammatory and insulin/IGF-1 pathways (12, 17). However, previous studies have mostly focused on total protein intake, and in-depth analyses of the impact of protein quality and its food sources remain limited. To fill this gap, we conducted a large-scale prospective cohort study to systematically explore the associations between protein quality and lung cancer incidence, mortality, and pathological subtypes after adjusting for various lifestyle and demographic confounding factors. This study is the first to find a significant negative correlation between the HPPQI and both lung cancer incidence and mortality risks, indicating that a high-HPPQI dietary pattern helps reduce lung cancer risk.

The HPPQI comprehensively evaluates dietary protein quality, considering diversity, essential amino acid composition, bioavailability, fat/cholesterol content, and micronutrient density. These factors together affect protein's physiological utilization and health benefits (19, 20). This study found a significant link between a high HPPQI and lower lung cancer incidence and mortality risks. Based on prior evidence, several potential mechanisms have been proposed that could explain the observed association. Micronutrients like selenium and vitamin D enhance the antioxidant defense system to resist environmental oxidative stress. High - quality protein and anti - inflammatory components (such as ω - 3 fatty acids) inhibit chronic inflammation. They also promote alveolar structural repair and lung parenchymal integrity. Dietary fiber from plant - based protein sources helps excrete exogenous carcinogens, reducing systemic toxin exposure (17, 37, 38). Although the HPPQI does not explicitly distinguish between animal- and plant-based protein sources, its scoring criteria essentially favor plant proteins and low-fat animal proteins, which is consistent with current evidence in nutritional epidemiology regarding high-quality proteins and food synergy. In the future, the assessment of different protein source types and their proportions could be more comprehensively integrated into dietary indices. This would enable a more precise representation of how overall dietary patterns contribute to lung cancer prevention. The findings of this study align with current epidemiological evidence, emphasizing that in the context of lung cancer prevention, it is crucial to prioritize protein quality over mere quantity (39). Future research ought to formulate more tailored dietary protein recommendations. This can be achieved by taking into account the genetic background associated with amino acid metabolism and individual exposure characteristics. By doing so, we can further unlock the application potential of the HPPQI in the realm of tumor nutrition prevention.

This study has limitations requiring attention. First, a major limitation of this study is the reliance on a single baseline dietary assessment. Given that dietary habits evolve over time, this approach may fail to capture the true cumulative impact of diet on disease incidence (40). Although baseline assessments generally reflect habitual long-term intake based on nutritional principles, the relatively brief DHQ used here likely underestimates dietary variability. More critically, this single-time measurement introduces the risk of non-differential misclassification, which tends to bias observed associations toward the null (i.e., masking true associations). These methodological constraints necessitate careful interpretation of our findings and highlight the need for future studies incorporating repeated dietary assessments to validate our results. Second, like most observational studies, residual confounding from unmeasured factors can't be entirely excluded. Third, self - reported dietary questionnaires are prone to recall bias, affecting the accuracy of dietary data. Fourth, though the study included a large sample of middle - aged and older adult(s) Americans, the HPPQI - lung cancer incidence/mortality relationship in other regions or age groups is unclear. Further research is needed to explore these associations across populations and identify subgroup differences. Fifth, this study did not incorporate important variables such as treatment types (surgery ± chemotherapy/radiotherapy or no treatment) and TNM staging information. The primary reason is that the PLCO database lacks relevant treatment and staging information. Given that treatment modalities and TNM staging are core factors influencing lung cancer prognosis, these limitations may introduce certain biases in the prediction results of OS and disease - free survival. Future research that integrates clinical databases containing detailed treatment information and TNM staging data will enable a more comprehensive understanding of prognostic factors. Sixth, the HPPQI is a ratio of preferred protein sources to red/processed meats and cheese, and thus captures a broader dietary pattern rather than protein quality in isolation. Consequently, the observed associations may be partly explained by other dietary components that covary with HPPQI, such as dietary fiber and fat composition. Future studies with more detailed dietary assessments are needed to disentangle the specific contributions of protein quality vs. overall dietary patterns. Seventh, despite comprehensive adjustment for smoking status, daily cigarette consumption, and pack-years of smoking (in sensitivity analyses), residual confounding by smoking cannot be fully ruled out, as participants with higher HPPQI tended to have lower smoking intensity. Moreover, the PLCO trial did not collect data on time since smoking cessation or passive smoking exposure, which may have further contributed to residual confounding. Eighth, participants with higher HPPQI tended to have not only lower smoking intensity but also higher education levels, lower BMI, and fewer chronic respiratory diseases. These factors collectively point to a generally healthier lifestyle profile. Therefore, the observed protective association may partly reflect broader health-conscious behaviors rather than an independent effect of HPPQI. Previous studies on dietary patterns and lung cancer risk have similarly emphasized the challenge of disentangling specific dietary components from overall lifestyle factors (41–43). Ninth, while we have adjusted for these factors, residual confounding by unmeasured or imperfectly measured dietary factors cannot be fully excluded. Finally, due to the study's observational nature, caution is warranted when making causal inferences about diet-cancer links. Furthermore, although we have conducted comprehensive adjustments, we do not deny any limitations of historical data. Participants excluded from the final analysis due to missing dietary data, incomplete questionnaires, or pre-existing cancer may differ from those included in terms of dietary habits, smoking behavior, and health status, and we cannot fully rule out the possibility of selection bias. Despite the fact that the biological associations between diet and diseases may be relatively stable, when extrapolating our study findings to the current population, it is necessary to consider changes in social, environmental, and clinical practices, such as the widespread use of low-dose CT screening. Consequently, the generalizability of the study results may be affected by factors including innovations in lung cancer screening technologies, advancements in treatment methods (e.g., targeted and immunotherapy), changes in smoking rates among the population over the past few decades, as well as potential differences between excluded and included participants. Furthermore, the PLCO database lacks TNM staging and treatment information, both of which strongly influence lung cancer mortality. Therefore, our mortality findings should be interpreted as supporting the primary incidence results rather than providing independent evidence for improved prognosis, and we cannot determine whether the observed mortality association reflects reduced incidence, better post-diagnosis survival, or both. Caution is therefore warranted when interpreting the generalizability of our findings.

This study has notable strengths supporting its findings. Data come from a large, prospective US cohort with over 100,000 participants of diverse occupations, recruited from 10 centers. The large, heterogeneous sample ensures high representativeness, and long - term follow - up enhances result reliability. The PLCO study's prospective design and sensitivity analyses minimize reverse causation from subclinical pathologies altering diets, boosting the credibility of diet - lung cancer associations. Selection bias is addressed by maintaining similar lung cancer diagnosis proportions in excluded and included groups, strengthening internal validity. After adjusting for multiple confounders, conclusions are robust (44). This is the first systematic exploration of the HPPQI - lung cancer incidence/mortality link, covering NSCLC and SCLC, offering new key evidence. Findings show a high - HPPQI diet reduces lung cancer incidence and mortality risks, holding after sensitivity analyses, emphasizing result reliability and significance.

From a public health perspective, a diet characterized by higher HPPQI—centered on diverse plant proteins (legumes, whole grains, nuts) with fish and lean poultry preferred over red/processed meats—aligns with current dietary guidelines for chronic disease prevention. However, given the observational design of this single study and the substantial changes in lung cancer screening, treatment, and smoking patterns since the PLCO cohort was recruited, these findings should be interpreted with caution when extrapolating to current populations. Future studies in contemporary cohorts are needed to validate our results before they can directly inform updated dietary guidelines.

## Conclusion

Considering the existing evidence and the intricate interplay between diet, physical activity, and body composition, diets characterized by higher HPPQI should be viewed as an integral part of a healthy lifestyle rather than an isolated risk factor. In brief, this study highlights a significant correlation between increased HPPQI and lower lung cancer incidence and mortality. These findings provide supportive evidence that a high-HPPQI dietary pattern may contribute to lung cancer prevention. Nevertheless, as the PLCO cohort was recruited decades ago—with subsequent changes in low-dose CT screening, treatment modalities, smoking prevalence, and dietary patterns—extrapolation of our findings to current populations requires caution. Future prospective studies in contemporary cohorts are needed to confirm these associations. Additionally, further research is required to isolate the specific contribution of protein quality *per se* from the broader dietary pattern reflected by HPPQI.

## Data Availability

The original contributions presented in the study are included in the article/Supplementary material, further inquiries can be directed to the corresponding authors.
