# Supplementary material for: Adherence to a diet with higher protein quality significantly reduces the risk of lung cancer: results from a population-based prospective study
Source: Front Public Health. 2026 Jul 8;14:1869028. doi: 10.3389/fpubh.2026.1869028 (PMC13388812; doi:10.3389/fpubh.2026.1869028)
Supplement: Supplementary file 1 [file Table_1.docx]

**SUPPLEMENTARY MATERIAL**

**Adherence to a diet with higher protein quality significantly reduces the risk of lung cancer: Results from a population-based prospective study.**

**Supplementary Table 1**. Distribution of variables with missing data before and after imputation.

| **Variable** | **Before imputation** | **After imputation** | **Number (%) with missing data** |
| --- | --- | --- | --- |
| **Race** |  |  | 37(0.04%) |
| white | 94,029 (92.4%) | 94,066 (92.4%) |  |
| non-white | 7,689 (7.6%) | 7,689 (7.6%) |  |
| **Education level** |  |  | 204(0.20%) |
| college below | 64,749 (63.8%) | 64,953 (63.8%) |  |
| college graduate | 17,848 (17.6%) | 17,848 (17.6%) |  |
| postgraduate | 18,954 (18.7%) | 18,954 (18.6%) |  |
| **Marriage** |  |  | 193(0.19%) |
| married | 79,633 (78.4%) | 79,826 (78.5%) |  |
| unmarried | 21,929 (21.6%) | 21,929 (21.5%) |  |
| **Diabetes history** |  |  | 538(0.53%) |
| no | 94,411 (93.3%) | 94,949 (93.3%) |  |
| yes | 6,806 (6.7%) | 6,806 (6.7%) |  |
| **Aspirin use history** |  |  | 444(0.44%) |
| no | 53,509 (52.8%) | 53,953 (53.0%) |  |
| yes | 47,802 (47.2%) | 47,802 (47.0%) |  |
| **X-ray_history** |  |  | 23(0.02%) |
| no | 46,280 (45.5%) | 46,303 (45.5%) |  |
| once | 32,918 (32.3%) | 32,918 (32.3%) |  |
| more than once | 18377(18.1%) | 18377(18.1%) |  |
| possibly | 4157(4.1%) | 4157(4.1%) |  |
| **Family history of lung cancer** |  |  | 781(0.77%) |
| no | 87,957 (87.1%) | 88,738 (87.2%) |  |
| yes | 10,569 (10.5%) | 10,569 (10.4%) |  |
| possibly | 2,448 (2.4%) | 2,448 (2.4%) |  |
| **Chronic bronchitis history** |  |  | 577(0.57%) |
| no | 96,846 (95.7%) | 97,423 (95.7%) |  |
| yes | 4,332 (4.3%) | 4,332 (4.3%) |  |
| **Emphysema history** |  |  | 533(0.53%) |
| no | 99,078 (97.9%) | 99,611 (97.9%) |  |
| yes | 2,144 (2.1%) | 2,144 (2.1%) |  |
| **Hypertension history** |  |  | 513(0.51%) |
| no | 68,194 (67.4%) | 68,707 (67.5%) |  |
| yes | 33,048 (32.6%) | 33,048 (32.5%) |  |
| **Family history of cancer** |  |  | 288(0.28%) |
| no | 44,611 (44.0%) | 44,899 (44.1%) |  |
| yes | 56,856 (56.0%) | 56,856 (55.9%) |  |
| **Smoking status** |  |  | 20(0.02%) |
| no | 48,560 (47.7%) | 48,580 (47.7%) |  |
| current/former | 53,175 (52.3%) | 53,175 (52.3%) |  |
| **Body mass index at baseline (kg/m2)** | 27.23±4.82 | 27.22±4.79 | 1348(1.34%) |
| **Weight fluctuation**^a^ | 2.88±0.76 | 2.84±0.82 | 1348(1.34%) |
| **Smoking pack-years** | 17.83±26.69 | 17.65±26.59 | 1164(1.16%) |
| **Physical activity level (min/day)** | 124.98±123.64 | 116.02±107.73 | 26059(25.60%) |
| **Daily cigarette consumption** |  |  | 125(0.12%) |
| 0 | 48,560 (47.8%) | 48,685 (47.9%) |  |
| 1-20 | 33,218 (32.7%) | 33,218 (32.6%) |  |
| >20 | 19,852 (19.5%) | 19,852 (19.5%) |  |

Note: Descriptive statistics are presented as (mean ± standard deviation) and number (percentage) for continuous and categorical.

^a^ Weight fluctuation defined as the participant's baseline weight minus weight at age 20.

**Supplementary Table 2**. Subgroup analyses between HPPQI and lung cancer incidence.

| **Variables** | **Number of participates** | | **Number of cases** | | **P_interaction_** |  | **HR** ^b^ **( 95% confidence interval )** | | | **P_trend_ (Q4 vs Q1)** |
| --- | --- | --- | --- | --- | --- | --- | --- | --- | --- | --- |
|  |  |  |  |  |  | **Quartile 1** | **Quartile 2** | **Quartile 3** | **Quartile 4** |  |
| **Age(years)** |  |  | | 0.040 | |  |  |  |  |  |
| <=65 | 71,872 | 993 | |  | | 1.00 (reference) | 0.72 (0.61, 0.85) | 0.70 (0.58, 0.83) | 0.55 (0.45, 0.67) | <0.001 |
| >65 | 29,883 | 713 | |  | | 1.00 (reference) | 0.88 (0.72, 1.07) | 0.91 (0.74, 1.12) | 0.74 (0.59, 0.92) | 0.013 |
| **Sex** |  |  | | 0.147 | |  |  |  |  |  |
| male | 49,496 | 1,012 | |  | | 1.00 (reference) | 0.78 (0.67, 0.91) | 0.83 (0.70, 0.98) | 0.55 (0.44, 0.68) | <0.001 |
| female | 52,259 | 694 | |  | | 1.00 (reference) | 0.76 (0.61, 0.94) | 0.70 (0.56, 0.87) | 0.65 (0.52, 0.80) | 0.002 |
| **Race** |  |  | | 0.503 | |  |  |  |  |  |
| white | 94,066 | 1,589 | |  | | 1.00 (reference) | 0.76 (0.67, 0.87) | 0.76 (0.66, 0.87) | 0.60 (0.51, 0.70) | <0.001 |
| non-white | 7,689 | 117 | |  | | 1.00 (reference) | 0.88 (0.49, 1.60) | 0.91 (0.51, 1.63) | 0.89 (0.52, 1.53) | 0.801 |
| **Marriage** |  |  | | 0.423 | |  |  |  |  |  |
| married | 79,826 | 1,245 | |  | | 1.00 (reference) | 0.75 (0.65, 0.86) | 0.77 (0.66, 0.90) | 0.58 (0.49, 0.69) | <0.001 |
| non-married | 21,929 | 461 | |  | | 1.00 (reference) | 0.82 (0.64, 1.06) | 0.73 (0.56, 0.95) | 0.67 (0.49, 0.92) | 0.016 |
| **Hypertension history** |  |  | | 0.771 | |  |  |  |  |  |
| no | 68,707 | 1,145 | |  | | 1.00 (reference) | 0.75 (0.64, 0.87) | 0.74 (0.63, 0.87) | 0.60 (0.50, 0.72) | <0.001 |
| yes | 33,048 | 561 | |  | | 1.00 (reference) | 0.81 (0.65, 1.00) | 0.79 (0.63, 1.00) | 0.64 (0.49, 0.82) | 0.002 |
| **Diabetes history** |  |  | | 0.528 | |  |  |  |  |  |
| no | 94,949 | 1,587 | |  | | 1.00 (reference) | 0.76 (0.67, 0.87) | 0.74 (0.65, 0.85) | 0.61 (0.53, 0.71) | <0.001 |
| yes | 6,806 | 119 | |  | | 1.00 (reference) | 0.85 (0.53, 1.37) | 1.10 (0.68, 1.76) | 0.65 (0.35, 1.19) | 0.221 |
| **Smoking status** |  |  | | 0.136 | |  |  |  |  |  |
| current/former | 53,175 | 1,565 | |  | | 1.00 (reference) | 0.76 (0.67, 0.87) | 0.75 (0.65, 0.86) | 0.58 (0.50, 0.68) | <0.001 |
| no | 48,580 | 141 | |  | | 1.00 (reference) | 0.91 (0.54, 1.54) | 1.02 (0.62, 1.68) | 1.06 (0.64, 1.75) | 0.641 |
| **Emphysema history** |  |  | | 0.009 | |  |  |  |  |  |
| no | 99,611 | 1,535 | |  | | 1.00 (reference) | 0.72 (0.63, 0.82) | 0.72 (0.63, 0.83) | 0.61 (0.52, 0.71) | <0.001 |
| yes | 2,144 | 171 | |  | | 1.00 (reference) | 1.30 (0.90, 1.89) | 1.19 (0.79, 1.80) | 0.54 (0.30, 0.96) | 0.023 |
| **Chronic bronchitis history** |  |  | | 0.056 | |  |  |  |  |  |
| no | 97,423 | 1,550 | |  | | 1.00 (reference) | 0.75 (0.66, 0.86) | 0.78 (0.68, 0.89) | 0.63 (0.54, 0.74) | <0.001 |
| yes | 4,332 | 156 | |  | | 1.00 (reference) | 0.93 (0.63, 1.35) | 0.58 (0.36, 0.93) | 0.41 (0.23, 0.71) | 0.001 |
| **BMI**^a^ |  |  | | 0.911 | |  |  |  |  |  |
| <=30 | 78,598 | 1,385 | |  | | 1.00 (reference) | 0.76 (0.66, 0.87) | 0.75 (0.65, 0.87) | 0.62 (0.53, 0.73) | <0.001 |
| >30 | 23,157 | 321 | |  | | 1.00 (reference) | 0.78 (0.58, 1.03) | 0.82 (0.61, 1.11) | 0.63 (0.44, 0.91) | 0.025 |
| **Aspirin use history** |  |  | | 0.970 | |  |  |  |  |  |
| no | 53,953 | 850 | |  | | 1.00 (reference) | 0.78 (0.65, 0.93) | 0.79 (0.66, 0.96) | 0.64 (0.52, 0.78) | <0.001 |
| yes | 47,802 | 856 | |  | | 1.00 (reference) | 0.75 (0.63, 0.90) | 0.73 (0.61, 0.88) | 0.59 (0.48, 0.73) | <0.001 |
| **Family history of lung cancer** |  |  | | 0.824 | |  |  |  |  |  |
| no | 88,738 | 1,348 | |  | | 1.00 (reference) | 0.77 (0.67, 0.89) | 0.78 (0.67, 0.90) | 0.63 (0.54, 0.75) | <0.001 |
| yes/possibly | 13,017 | 358 | |  | | 1.00 (reference) | 0.74 (0.57, 0.97) | 0.70 (0.52, 0.93) | 0.54 (0.39, 0.74) | 0.001 |
| **Daily cigarette consumption** |  |  | | 0.204 | |  |  |  |  |  |
| 0 | 48,685 | 145 | |  | | 1.00 (reference) | 0.91 (0.54, 1.51) | 1.00 (0.61, 1.63) | 1.02 (0.62, 1.66) | 0.782 |
| 1-20 | 33,218 | 701 | |  | | 1.00 (reference) | 0.73 (0.60, 0.89) | 0.72 (0.59, 0.89) | 0.64 (0.52, 0.80) | 0.001 |
| >20 | 19,852 | 860 | |  | | 1.00 (reference) | 0.78 (0.66, 0.93) | 0.77 (0.64, 0.93) | 0.52 (0.41, 0.65) | <0.001 |
| **History of alcohol consumption** |  |  | | 0.339 | |  |  |  |  |  |
| no | 27,757 | 435 | |  | | 1.00 (reference) | 0.74 (0.57, 0.97) | 0.89 (0.69, 1.16) | 0.72 (0.55, 0.94) | 0.056 |
| yes | 73,998 | 1271 | |  | | 1.00 (reference) | 0.77 (0.66, 0.88) | 0.72 (0.62, 0.84) | 0.58 (0.48, 0.69) | <0.001 |

^a^ BMI was defined as body mass index at baseline (kg/m2).

^b^ Hazard ratio was adjusted for age (years), sex (male, female), race (white and non-white), education levels (college below, college graduate, postgraduate), marital status (married, unmarried), smoking status (never, currently/ever), number of cigarettes smoked (0, 1-20, > 20 cigarettes/day), history of alcohol consumption (yes, no), physical activity level (min/day) , history of emphysema (yes, no), chronic bronchitis history (yes, no), body mass index (kg/m2), trail arm (intervention, control), aspirin use (yes, no), history of diabetes (yes, no), history of hypertension (yes, no) and family history of lung cancer (yes, no).

**Supplementary Table 3**. Subgroup analyses between HPPQI and lung cancer mortality.

| **Variables** | | **Number of participates** | | | **Number of cases** | | **P_interaction_** |  | | **HR**^b^ **( 95% confidence interval )** | | | | | | **P_trend_ (Q4 vs Q1)** | |
| --- | --- | --- | --- | --- | --- | --- | --- | --- | --- | --- | --- | --- | --- | --- | --- | --- | --- |
|  |  |  |  |  |  |  |  | **Quartile 1** | | **Quartile 2** | | **Quartile 3** | | **Quartile 4** | |  |  |
| **Age(years)** | |  |  | | 0.033 | | |  | |  | |  | |  | |  | |
| <=65 | | 71,872 | 691 | |  | | | 1.00 (reference) | | 0.70 (0.58, 0.85) | | 0.69 (0.56, 0.85) | | 0.54 (0.42, 0.68) | | <0.001 | |
| >65 | | 29,883 | 526 | |  | | | 1.00 (reference) | | 1.03 (0.82, 1.29) | | 0.97 (0.76, 1.23) | | 0.74 (0.57, 0.97) | | 0.013 | |
| **Sex** | |  |  | | 0.405 | | |  | |  | |  | |  | |  | |
| male | | 49,496 | 753 | |  | | | 1.00 (reference) | | 0.81 (0.68, 0.97) | | 0.84 (0.70, 1.03) | | 0.57 (0.44, 0.74) | | <0.001 | |
| female | | 52,259 | 464 | |  | | | 1.00 (reference) | | 0.80 (0.62, 1.05) | | 0.68 (0.53, 0.89) | | 0.61 (0.47, 0.79) | | 0.001 | |
| **Race** | |  |  | | 0.243 | | |  | |  | |  | |  | |  | |
| white | | 94,066 | 1,137 | |  | | | 1.00 (reference) | | 0.80 (0.69, 0.93) | | 0.76 (0.65, 0.89) | | 0.57 (0.48, 0.69) | | <0.001 | |
| non-white | | 7,689 | 80 | |  | | | 1.00 (reference) | | 1.12 (0.53, 2.39) | | 1.19 (0.57, 2.47) | | 1.15 (0.58, 2.29) | | 0.818 | |
| **Marriage** | |  |  | | 0.677 | | |  | |  | |  | |  | |  | |
| married | | 79,826 | 882 | |  | | | 1.00 (reference) | | 0.78 (0.66, 0.93) | | 0.76 (0.63, 0.91) | | 0.57 (0.46, 0.70) | | <0.001 | |
| non-married | | 21,929 | 335 | |  | | | 1.00 (reference) | | 0.89 (0.66, 1.20) | | 0.82 (0.61, 1.11) | | 0.69 (0.51, 0.95) | | 0.026 | |
| **Hypertension history** | |  |  | | 0.425 | | |  | |  | |  | |  | |  | |
| no | | 68,707 | 832 | |  | | | 1.00 (reference) | | 0.76 (0.64, 0.91) | | 0.71 (0.59, 0.86) | | 0.58 (0.47, 0.72) | | <0.001 | |
| yes | | 33,048 | 385 | |  | | | 1.00 (reference) | | 0.90 (0.70, 1.17) | | 0.90 (0.69, 1.19) | | 0.64 (0.47, 0.88) | | 0.006 | |
| **Diabetes history** | |  |  | | 0.741 | | |  | |  | |  | |  | |  | |
| no | | 94,949 | 1,123 | |  | | | 1.00 (reference) | | 0.82 (0.70, 0.95) | | 0.76 (0.65, 0.89) | | 0.60 (0.50, 0.72) | | <0.001 | |
| yes | | 6,806 | 94 | |  | | | 1.00 (reference) | | 0.71 (0.42, 1.21) | | 0.92 (0.54, 1.56) | | 0.56 (0.28, 1.12) | | 0.164 | |
| **Smoking status** | |  |  | | 0.276 | | |  | |  | |  | |  | |  | |
| current/former | | 53,175 | 1,124 | |  | | | 1.00 (reference) | | 0.81 (0.70, 0.94) | | 0.75 (0.64, 0.89) | | 0.57 (0.48, 0.69) | | <0.001 | |
| no | | 48,580 | 93 | |  | | | 1.00 (reference) | | 0.80 (0.42, 1.53) | | 1.09 (0.60, 1.97) | | 0.99 (0.54, 1.83) | | 0.811 | |
| **Emphysema history** | |  |  | | 0.019 | | |  | |  | |  | |  | |  | |
| no | | 99,611 | 1,098 | |  | | | 1.00 (reference) | | 0.76 (0.65, 0.89) | | 0.73 (0.62, 0.87) | | 0.61 (0.51, 0.73) | | <0.001 | |
| yes | | 2,144 | 119 | |  | | | 1.00 (reference) | | 1.31 (0.84, 2.04) | | 1.18 (0.73, 1.93) | | 0.38 (0.18, 0.83) | | 0.010 | |
| **Chronic bronchitis history** | |  |  | | 0.281 | | |  | |  | |  | |  | |  | |
| no | | 97,423 | 1,112 | |  | | | 1.00 (reference) | | 0.80 (0.69, 0.94) | | 0.79 (0.67, 0.93) | | 0.62 (0.52, 0.75) | | <0.001 | |
| yes | | 4,332 | 105 | |  | | | 1.00 (reference) | | 0.86 (0.54, 1.37) | | 0.57 (0.32, 1.00) | | 0.36 (0.18, 0.72) | | 0.003 | |
| **BMI**^a^ | |  |  | | 0.821 | | |  | |  | |  | |  | |  | |
| <=30 | | 78,598 | 990 | |  | | | 1.00 (reference) | | 0.79 (0.67, 0.93) | | 0.75 (0.63, 0.90) | | 0.61 (0.50, 0.74) | | <0.001 | |
| >30 | | 23,157 | 227 | |  | | | 1.00 (reference) | | 0.85 (0.61, 1.19) | | 0.86 (0.60, 1.23) | | 0.61 (0.39, 0.94) | | 0.032 | |
| **Aspirin use history** | |  |  | | 0.848 | | |  | |  | |  | |  | |  | |
| no | | 53,953 | 610 | |  | | | 1.00 (reference) | | 0.85 (0.69, 1.04) | | 0.83 (0.66, 1.03) | | 0.64 (0.50, 0.82) | | 0.001 | |
| yes | | 47,802 | 607 | |  | | | 1.00 (reference) | | 0.77 (0.63, 0.95) | | 0.72 (0.58, 0.89) | | 0.56 (0.44, 0.72) | | <0.001 | |
| **Family history of lung cancer** | |  |  | | 0.632 | | |  | |  | |  | |  | |  | |
| no | | 88,738 | 958 | |  | | | 1.00 (reference) | | 0.81 (0.69, 0.96) | | 0.79 (0.66, 0.94) | | 0.64 (0.53, 0.78) | | <0.001 | |
| yes/possibly | | 13,017 | 259 | |  | | | 1.00 (reference) | | 0.78 (0.57, 1.06) | | 0.72 (0.52, 1.01) | | 0.46 (0.31, 0.69) | | <0.001 | |
| **Daily cigarette consumption** | |  |  | | 0.273 | | |  | |  | |  | |  | |  | |
| 0 | | 48,685 | 95 | |  | | | 1.00 (reference) | | 0.78 (0.41, 1.49) | | 1.10 (0.62, 1.97) | | 0.97 (0.53, 1.77) | | 0.869 | |
| 1-20 | | 33,218 | 495 | |  | | | 1.00 (reference) | | 0.79 (0.62, 1.00) | | 0.71 (0.55, 0.91) | | 0.65 (0.50, 0.85) | | 0.006 | |
| >20 | | 19,852 | 627 | |  | | | 1.00 (reference) | | 0.82 (0.68, 1.00) | | 0.78 (0.63, 0.97) | | 0.49 (0.37, 0.64) | | <0.001 | |
| **History of alcohol consumption** | |  |  | | 0.057 | | |  | |  | |  | |  | |  | |
| no | | 27,757 | 307 | |  | | | 1.00 (reference) | | 0.90 (0.66, 1.23) | | 1.11 (0.82, 0.50) | | 0.68 (0.49, 0.96) | | 0.028 | |
| yes | | 73,998 | 910 | |  | | | 1.00 (reference) | | 0.78 (0.66, 0.92) | | 0.68 (0.56, 0.81) | | 0.58 (0.47, 0.71) | | <0.001 | |

^a^ BMI was defined as body mass index at baseline (kg/m2).

^b^ Hazard ratio was adjusted for age (years), sex (male, female), race (white and non-white), education levels (college below, college graduate, postgraduate), marital status (married, unmarried), smoking status (never, currently/ever), number of cigarettes smoked (0, 1-20, > 20 cigarettes/day), history of alcohol consumption (yes, no), physical activity level (min/day) , history of emphysema (yes, no), chronic bronchitis history (yes, no), body mass index (kg/m2), trail arm (intervention, control), aspirin use (yes, no), history of diabetes (yes, no), history of hypertension (yes, no) and family history of lung cancer (yes, no).
